# Editorial: Endocrine disruptors affecting the human and companion animal endocrine functions – similarities and indicators in ONE Health concept

**DOI:** 10.3389/fendo.2023.1324986

**Published:** 2023-10-31

**Authors:** Julianna Thuróczy

**Affiliations:** Gamma-Vet Ltd, Animal Health Center Budafok, Budapest, Hungary

**Keywords:** environment, endocrine disruptor (EDC), One Health, phthalates, toxicology

The effects of environmental pollution and climate change, often referred to as the ‘evils of civilization,’ both have an impact on metabolism and the emergence of malignant diseases. The One Health concept is an approach based on the close connection among people, animals, and the environment, encompassing environmental hazards and zoonotic diseases, but also other threats shared by humans, animals, and the environment. In the modern world, the concept of One Health has gained significant attention. One Health recognises the interconnectedness of human, animal, and environmental health. It emphasises the idea that the health of these three domains is interdependent and that a holistic approach is required to address global health challenges. In these articles, researchers delve into the effects of endocrine disruptor chemicals (EDCs) on the One Health concept and demonstrate brilliant summary of their conclusion.

Puberty is a fundamental phase in development. Puberty initiation and completion are influenced by a multitude of factors, including genetics, epigenetics, lifestyle, stress, and energy balance. Uğurlu et al. investigate the relationship between blue light exposure and the increased incidence of precocious puberty, utilizing rats as an animal model that mimics human physiology. The findings suggest that blue light exposure triggers central puberty in both males and females. Blue light is pervasive in today’s society, especially among children. The study indicates that blue light may suppress the hormone leptin, increase appetite, and, in turn, lead to an increase in adipose tissue. The study found that blue light exposure led to smaller and lighter testicular tissue, with lower testosterone and DHEAS levels. Spermatogenesis, influenced by LH and FSH, appeared to be suppressed with extended blue light exposure. The study observed histological changes in testicular tissue due to blue light exposure, including damage to the seminiferous tubules. The findings highlight the potential adverse effects of excessive blue light exposure, a concern given the increasing reliance on electronic devices in our daily lives.

Phthalates, commonly used as plasticizers in various industrial products, have raised concerns due to their potential endocrine-disrupting effects on both humans and wildlife. Exposure to different types of phthalates has been linked to the development of various diseases. However, the specific impact of *in-utero* or oral exposure to two particularly concerning phthalates, DHP and DCHP, on liver metabolism had not been extensively studied. Aydemir et al. investigated the effects of *in-utero* exposure to DHP and DCHP on liver health. Liver function indicators showed significant changes in response to phthalate exposure. Histopathological examination of liver samples revealed various signs of liver damage, including congestion, sinusoidal dilatation, inflammatory cell infiltration, cell death, and degeneration of hepatic parenchyma. Evidence of the detrimental impact of *in-utero* exposure is provided to DHP and DCHP on the liver’s oxidative stress metabolism and function in male and female rats. These findings emphasize the need for stricter regulation and reduction of phthalate production, particularly among pregnant individuals. EDCs pose a significant risk to human health, resulting in various adverse effects, including reproductive dysfunction and metabolic disorders. Nevertheless, the oxidative stress induced by EDCs can be mitigated through antioxidant treatment, potentially reducing the risk of early and premature menopause (Aydemir and Ulusu).

Mao et al. explore the potential benefits of Platelet-Rich Fibrin (PRF) treatment in regenerating damaged endometrium. *In vivo* experiments involving PRF transplantation showed benefits for uterine structure maintenance and regeneration of the endometrial luminal epithelium and endometrial glands. PRF provided continuous and steady releases of various growth factors over two weeks, which may contribute to cell proliferation, angiogenesis, and cell migration, ultimately facilitating rapid tissue healing and regeneration. These growth factors are known to play key roles in endometrial regeneration. PRF is recognized for its matrix properties, including the presence of glycosaminoglycans that support cell migration and healing processes. The study found that PRF transplantation reduced neutrophil infiltration, which may have an anti-fibrotic effect by mitigating the release of neutrophil extracellular traps. The study concluded that PRF is effective in preventing intrauterine adhesions and promoting endometrial regeneration in a rat model, offering promise for women with poor endometrium.

Maric et al. delve into the impact of nickel (Ni) exposure on thyroid function in the general population. Traditionally, toxicological studies have primarily relied on animal testing, often involving high doses of substances that may not accurately reflect real-world environmental chemical exposures. This discrepancy is particularly notable when studying EDCs due to their ability to provoke potent effects at low doses and exhibit non-monotonic behaviour. The results suggest that Ni may interfere with apoptosis-related proteins and trigger oxidative stress in thyroid tissue, leading to alterations in hormone production. Differences in Ni levels were observed between genders, potentially due to hormonal disparities, metabolic rates, body composition, and lifestyle factors. Correlation analyses were conducted between Ni concentration and hormone levels and function parameters. While there is limited research exploring the effects of Ni on thyroid function, the study offers valuable insights. It highlights the potential importance of examining additional risk factors for thyroid disorders, particularly in regions with no iodine deficiency. The study suggests a dose-response relationship between Ni and thyroid function parameters. However, these relationships have relatively wide intervals, reflecting uncertainties inherent in human data analyses. While more research is needed, especially regarding Ni’s mechanisms of toxicity, this study opens the door to exploring the potential influence of Ni on thyroid function, particularly in males.

Ullah et al. reviewed highlights the profound and wide-ranging effects of microplastics (MPs), nanoplastics (NPs), and the associated chemicals on the endocrine systems of mammals. The chemical composition of MPs, which includes various plastic additives, is a cause for concern. The detrimental effects of MPs and NPs on specific endocrine organs, such as the thyroid gland, male reproductive system, female reproductive system, hypothalamus, and pituitary gland, are well-documented. Moreover, the “Trojan Horse” effect of MPs is a serious concern, as these particles serve as vehicles for the transportation of other toxic pollutants, such as endocrine-disrupting chemicals and heavy metals. This results in bioaccumulation and biomagnification in mammalian tissues, affecting the function of various organs and leading to metabolic disorders, gut dysbiosis, and intestinal barrier dysfunction. Additionally, MPs have been found in human feces, highlighting their presence in the human food chain and raising concerns about their potential impacts on human health. These effects range from disruptions in hormonal balance to structural and functional abnormalities in these organs, impacting fertility, development, and overall health. The preservation of our ecosystems and the protection of the endocrine systems that regulate the health and development of all living organisms depend on our collective commitment to addressing this pressing issue.

## Author contributions

JT: Writing – original draft.

